# Clinico-Immunological Profile of a 67-Year-Old Woman Affected by HER2-Positive Breast Cancer and Autoimmune Dermatomyositis

**DOI:** 10.3389/fonc.2020.00192

**Published:** 2020-02-25

**Authors:** Benedetta Pellegrino, Giulia Mazzaschi, Denise Madeddu, Cristina Mori, Costanza Anna Maria Lagrasta, Gabriele Missale, Federico Quaini, Antonino Musolino

**Affiliations:** ^1^Medical Oncology and Breast Unit, University Hospital of Parma, Parma, Italy; ^2^Department of Medicine and Surgery, University of Parma, Parma, Italy; ^3^Unit of Infectious Diseases and Hepatology, University Hospital of Parma, Parma, Italy; ^4^Pathology Unit, University Hospital of Parma, Parma, Italy; ^5^Gruppo Oncologico Italiano di Ricerca Clinica, Parma, Italy

**Keywords:** breast cancer, HER2, dermatomyositis, trastuzumab, autoimmune, cross-reactivity

## Abstract

A patient with HER2-positive early breast cancer (BC) developed dermatomyositis (DM), which disappeared after the first administration of adjuvant trastuzumab. No HER2 overexpression/amplification was observed in DM skin biopsies. Both BC and skin immune infiltrates were composed mostly of CD3+ T-lymphocytes. Interestingly, tumor-infiltrating lymphocytes expressed PD-1, which was negligible in skin-infiltrating lymphocytes, while both BC cells and keratinocytes were PD-L1-positive. High serum levels of endogenous anti-HER2 antibodies were detected, confirming the induction of a HER2-specific adaptive immune response. It may be argued that HER2-specific T-lymphocytes cross-reacted with one or more unknown skin antigens, causing DM. Trastuzumab may have silenced skin cross-reaction by eliminating any residual HER2-positive micrometastatic disease and, thus, inducing DM remission.

## Background

HER2 (Her-2/neu, c-erbB-2) is a 185-kDa transmembrane tyrosine kinase protein giving higher aggressiveness in breast cancers (BCs). In humans, HER2 overexpression occurs in 15–20% of primary breast tumors, and is associated with diminished disease-free (DFS) and overall survival (OS) (1). The humanized immunoglobulin G1 (IgG1) anti-HER2 monoclonal antibody (mAb) trastuzumab in combination with chemotherapy is an effective treatment for all stages of HER2-positive BC (2). Growing evidence suggests a clear role of the host immune system in HER2-positive BC, which is generally considered more immunogenic than other BC subgroups (3).

Dermatomyositis (DM) is an autoimmune disease consisting of a chronic inflammatory injury of striated muscle and skin with an incidence of 1/100,000 (4). It is usually associated with activation of auto-reactive T lymphocytes, down-regulation of T regulator cells and release of pro-inflammatory cytokines leading to B and T cells tolerance loss (5). DM patients can develop additional autoimmune diseases, and there is an elevated occurrence of other autoimmune diseases in close relatives (6). Genome-wide association studies (GWAS) have confirmed the MHC as the major genetic region associated with DM and have indicated that DM shares non-MHC genetic features with other autoimmune diseases, suggesting the presence of additional novel risk loci (6). Approximately 15–30% of DMs are associated with underlying malignancies [standardized incidence ratio (SIR) 3.0, 95% CI 2.5–3.6] (4, 5). In particular, DM has been strongly associated with ovarian (SIR 10.5, 95% CI 6.1–18.1), lung (5.9, 3.7–9.2), pancreatic (3.8, 1.6–9.0), stomach (3.5, 1.7–7.3), colorectal (2.5, 1.4–4.4), and breast cancers (2.2, 1.2–3.9) (6). The molecular mechanisms underlying these associations are still unknown, even though it has been demonstrated a possible antigenic similarity between regenerating myoblasts and some cancer cell populations (5–7). Here we report the clinical history of a patient with HER2-positive early BC who developed dermatomyositis (DM), which disappeared after the first administration of adjuvant trastuzumab. Biological and clinical implications of the treatment outcome observed in this case are discussed with the knowledge of scientific evidence to date available.

## Case Presentation

In November 2014, a 67-year-old woman with neither comorbidities nor personal or familial history for autoimmune diseases was diagnosed with a ductal carcinoma of the right breast. She underwent right quadrantectomy and sentinel node biopsy. Histology and immunohistochemistry (IHC) confirmed pT1c (2 cm) N0M0 infiltrating ductal carcinoma, grade 3, which resulted Estrogen Receptor (ER)-negative, Progesterone Receptor (PR)-negative, HER2-positive (3+ by IHC and FISH positive), and Ki67^high^ (50%). The quantitative assessment of tumor-infiltrating lymphocytes(TILs) documented intermediate (>5% and <50%) TIL infiltration (8).

**Figure 1:**
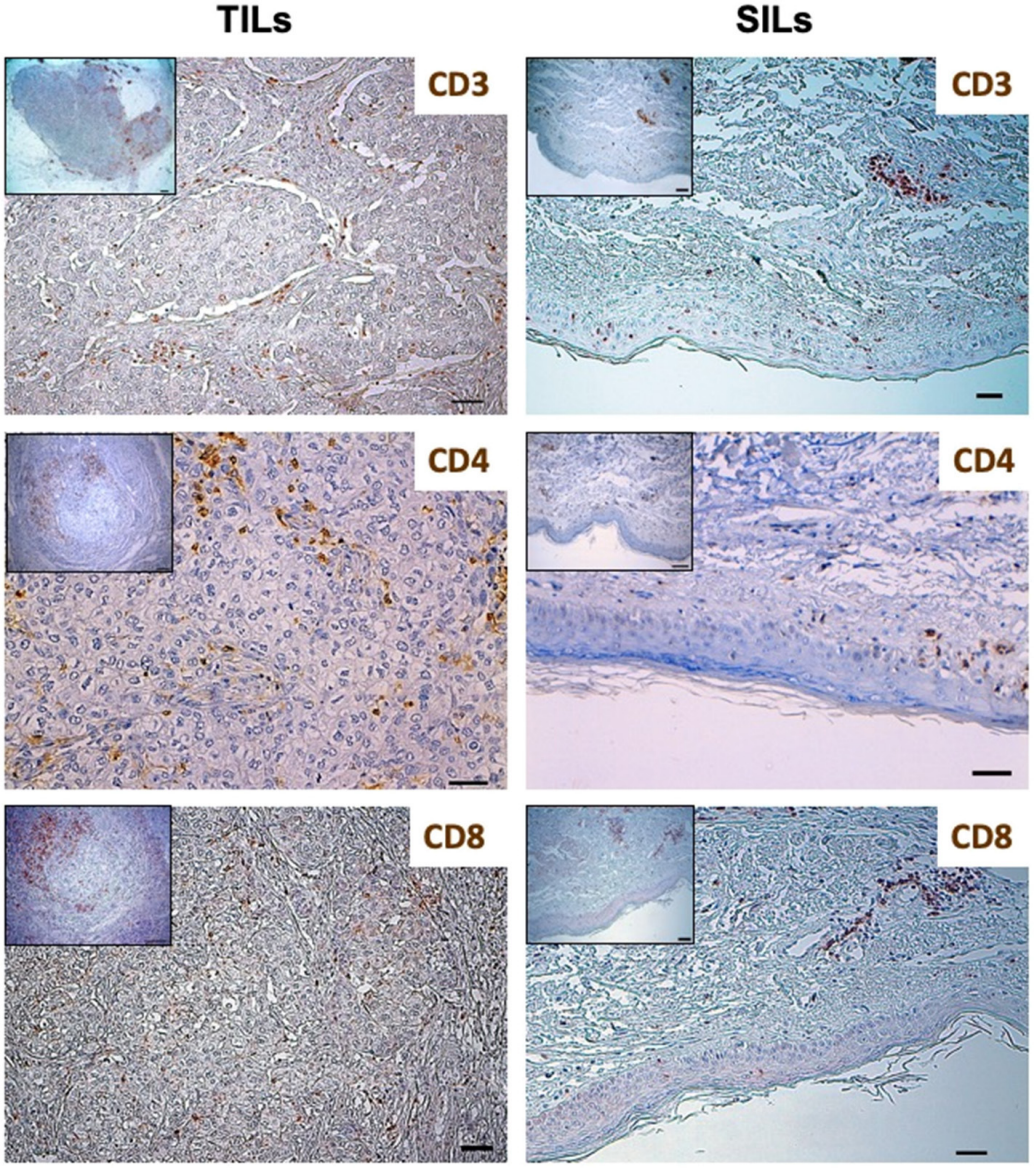
Distribution of lymphocytes subpopulations in Tumor-Infiltrating Lymphocytes (TILs) and Skin-Infiltrating Lymphocytes (SILs) by immunohistochemistry. Immunohistochemistry was performed on sections from breast cancer and skin to assess the incidence of CD3, CD8, and CD4 expressing cells. The axillary lymph node was used as a control for the tissue analysis of these subpopulations of lymphocytes. Samples were incubated with anti-CD3 (mouse monoclonal 1:100, 30′, 37°C, DAKO), anti-CD8 (rabbit monoclonal, clone SP16, 1:50, o/n a 4°C, NEOMARKERS), and anti-CD4 (mouse monoclonal, clone 4B12, 1:100, 30′, 37°C, DAKO) antibodies, followed by DAB Detection Kit (Ventana, Roche). Finally, sections were counterstained with Mayer's hematoxylin. The quantitative evaluation of CD3+, CD8+, and CD4+ TILs on breast cancer sample was performed considering their proximal (<20 μm) or distal (>20 μm) localization with respect to neoplastic cells (intratumoral or peritumoral TILs). Thus, the number per unit area (n/mm^2^) of tissue of each subpopulation of TILs was computed. This analysis involved a sampled area of 30 mm^2^ of tissue and counting from a minimum of 500 to a maximum of 1,700 cells for each epitope. All the analyses were performed in breast cancer and skin tissue samples obtained before treatment initiation. TILs and SILs were composed mostly of CD3+ T cells. The CD4/CD8 TIL ratio was 4.17; conversely, the phenotypic distribution of SILs documented the prevalence of CD8+ cells over the CD4+ subpopulation (CD4/CD8 SIL ratio: 0.74).

In January 2015, the patient was admitted to the University Hospital of Parma because of a 3-month history of intense and diffuse muscle pain and pruriginous erythema of the trunk, arms, and legs. Blood tests showed high serum levels of AST, ALT, CPK, and LDH. Electromyography and skin biopsy confirmed the diagnosis of dermatomyositis (DM). No clinical benefit was observed with steroids therapy (prednisone, 1 mg/kg/day for 1 month). As shown in Figure 1 and Table 1, the phenotypic distribution of skin-infiltrating lymphocytes (SILs) documented the prevalence of CD8+ T cells.

**Table 1:** Tissue distribution of lymphocyte subpopulations.

**Lymphocytes**	**CD3 n/mm^**2**^**	**CD8 n/mm^**2**^**	**PD-1 n/mm^**2**^**	**CD4 n/mm^**2**^**
TILs, intratumoral	55.11	9.89	19.26	9.36
TILs, peritumoral	24.26	3.09	2.66	12.77
TILs, total	79.37	12.98	21.92	22.13
SILs	62.77	58.24	16.49	43.22
BILs	79.04	17.87	9.57	36.17

*TILs, tumor-infiltrating lymphocytes; SILs, skin-infiltrating lymphocytes; BILs, breast tissue-infiltrating lymphocytes*.

### Trastuzumab-Based Adjuvant Treatment

In February 2015, the patient was admitted to the Breast Unit of the University Hospital of Parma to receive adjuvant chemo-immunotherapy (CIT) with trastuzumab (8 mg/kg IV loading dose, followed by 6 mg/kg), cyclophosphamide (600 mg/m^2^) and docetaxel (75 mg/m^2^) every 3 weeks for four cycles. Then, 14 cycles of single-agent trastuzumab (6 mg/Kg, q3w) were administered and radiotherapy (RT) on residual breast was planned after chemotherapy. Interestingly, after the first administration of CIT with trastuzumab, we observed the complete remission of DM.

To date, 42 months after the last administration of trastuzumab (February 2016), the patient is disease-free from both BC and DM. A new skin biopsy was performed in December 2016 confirming complete pathological remission of DM.

### Description of Laboratory Investigations

**Figure 2:**
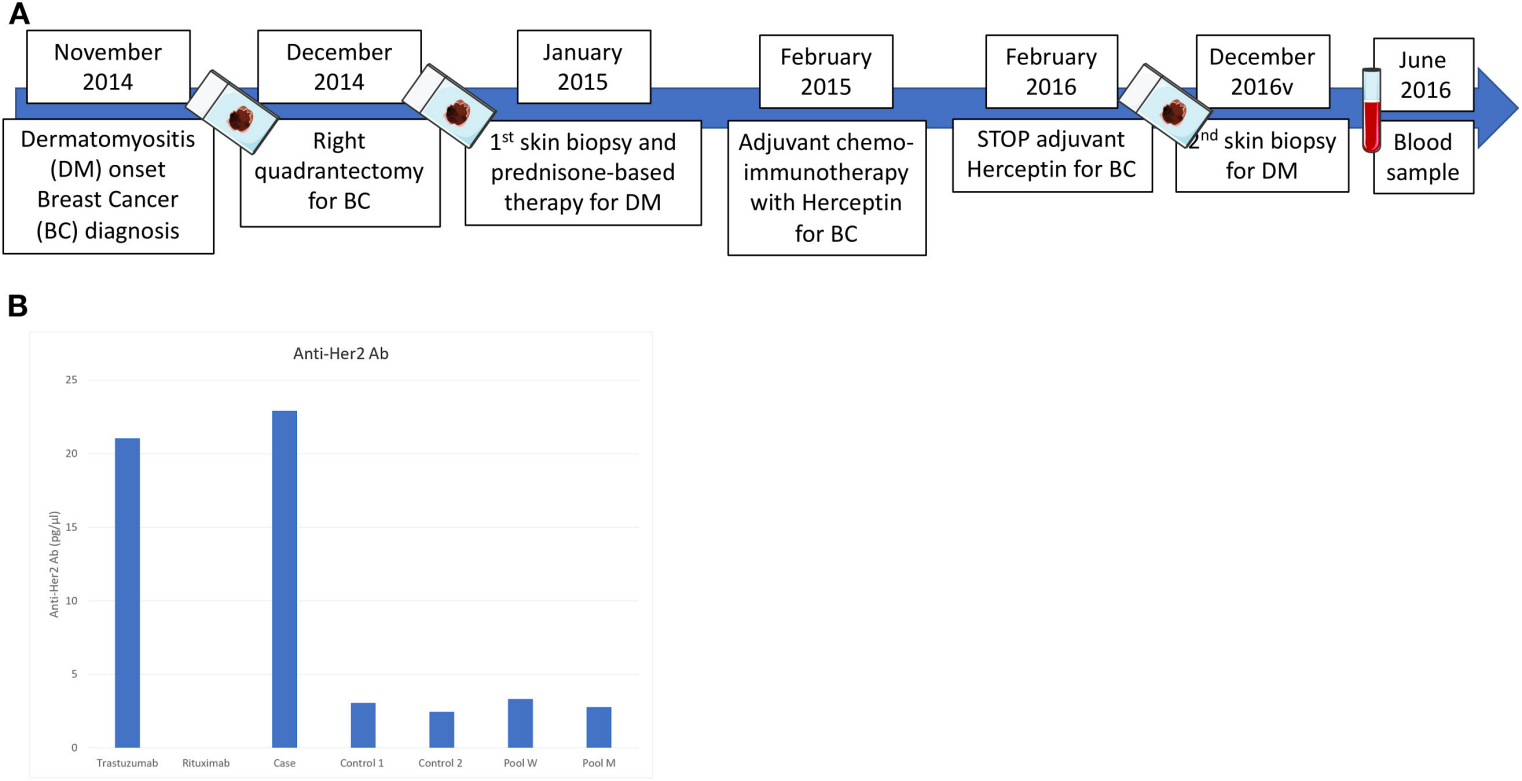
**(A)** Timeline diagram of blood, tumor and skin tissue investigations performed on the patient from diagnosis to follow-up; **(B)** Anti-HER2 antibodies serum concentration quantified by ELISA. Serum from two Her-2^positive^ early BC patients and pool serum from 10 healthy men (M) and 10 women (W), respectively, were used as comparison. Rituximab was used as negative control. After 16 months from the last administration of trastuzumab, the patient presented a serum concentration of anti-HER2 specific antibodies of 23.2 pg/μl, which was considerably higher compared to that observed in the control group.

After obtaining patient's written informed consent and independent ethics committee (IEC) approval, we performed additional serological and IHC analyses on blood and formalin fixed paraffin embedded (FFPE) samples of BC and DM (timeline diagram illustrated in Figure 2A).

To quantify the autologous immune response to HER2-positive BC, we determined the serum anti-HER2 antibody titers by ELISA. After 16 months from the last administration of trastuzumab, the patient presented a serum concentration of anti-HER2 specific antibodies of 23.2 pg/μl, which was considerably higher compared to that observed in a control group composed of HER2-positive BC patients at the same disease stage, and healthy volunteers (Figure 2B).

**Figure 3:**
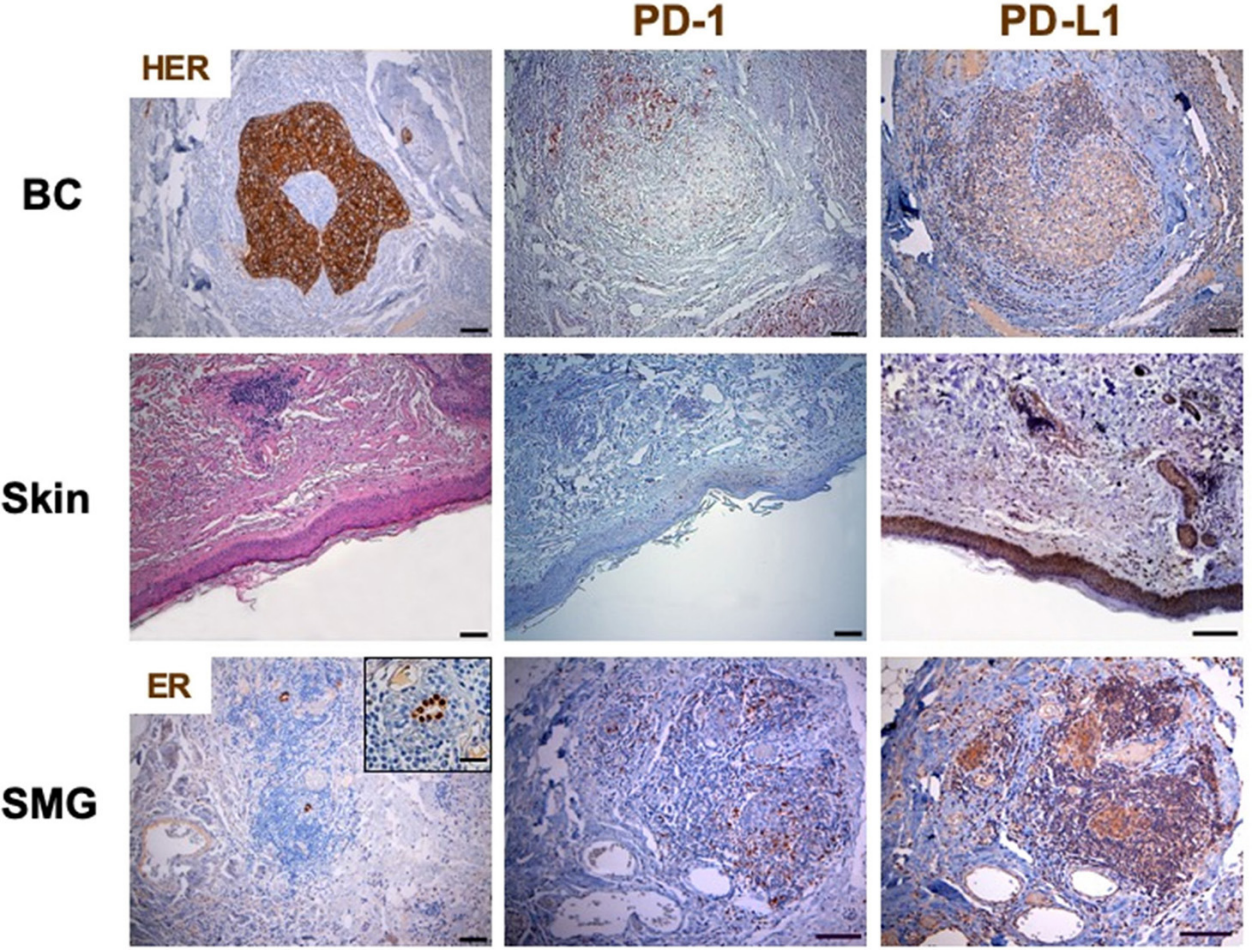
PD-1 and PD-L1 expression in breast cancer and skin by immunohistochemistry. Immunohistochemistry was performed on sections from breast cancer and skin to assess the incidence of PD-1 expressing cells. The axillary lymph node was used as a control for the tissue analysis of these subpopulations of lymphocytes. Samples were incubated with anti-PD-1 (mouse monoclonal, clone NAT105, 1:100, 30′, 37°C, ROCHE) antibody, followed by DAB Detection Kit (Ventana, Roche). Finally, sections were counterstained with Mayer's hematoxylin. The quantitative evaluation of PD-1-positive TILs on breast cancer sample was performed considering their proximal (<20 μm) or distal (>20 μm) localization with respect to neoplastic cells. Thus, the number per unit area (n/mm^2^) of tissue of each subpopulation of TILs was computed. PD-L1 was detected by immunoperoxidase on serial sections from breast cancer and skin using two different anti-PD-L1 antibodies (clones 28-8 and SP142). Sections from human placenta, considered as a reference tissue were similarly investigated. The best results in terms of specificity and sensitivity were obtained by incubating samples with the primary antibody anti PD-L1 clone 28-8 (rabbit monoclonal, 1:50, o/n a 4°C, Abcam). Following suitability criteria, PD-L1 expression was measured on the entire section of the tumor. An algorithm was used to obtain the PD-L1 score (H-score; 0–300), which is computed on the basis of both extent and intensity of PD-L1 staining. All the analyses were performed in breast cancer and skin tissue samples obtained before treatment initiation. No HER2 overexpression was observed in DM skin biopsies. In BC, PD-1 expressing immune cells, which were predominantly adjacent to neoplastic cells, exceeded the number of cytotoxic CD8+ lymphocytes (PD-1/CD8 intratumoral TILs ratio: 1.94). Conversely, although PD-1-positive immune cells were also observed in the skin, their prevalence was significantly lower than that measured in BC. PD-L1 was clearly detectable in more than 10% of BC cells, which showed a predominant diffuse pattern of expression. Normal, ER-positive, mammary gland at the periphery of the tumor mass also displayed PD-L1 overexpression. The same analysis on the skin biopsy, obtained at the time of overt DM, showed that PD-L1 was greatly expressed by the basal layer of epidermal cells and, to a more extent, by dermal adnexa. Similar to what observed in DM skin samples, most residual mammary tissue (SMG), located along the tumor invasive margin, was highly infiltrated by CD8+ T-lymphocytes and low expression of PD-1 (PD-1/CD8 ratio in SMG: 0.54). ER, estrogen receptor; SMG, spared mammary gland; TILs, tumor infiltrating lymphocytes; SILs, skin infiltrating lymhpocytes.

TILs were composed mostly of CD3+ T cells. To better define the T lymphocyte subpopulations engaged in cancer immune response, the density and distribution of CD3+, CD4+, CD8+ and programmed cell death-1 (PD-1)-positive TILs were measured (Figures 1, 3, Table 1). The CD4/CD8 TILs ratio was 4.17 and PD-1 expressing immune cells, which were predominantly adjacent to neoplastic cells, exceeded the number of cytotoxic CD8+ lymphocytes (PD-1/CD8 intratumoral TILs ratio: 1.94) (Figures 1, 3, Table 1). Conversely, the phenotypic distribution of SILs documented the prevalence of CD8+ cells over the CD4+ subpopulation (CD4/CD8 SILs ratio: 0.74) (Figure 1, Table 1). Although PD-1-positive immune cells were also observed in the skin, their prevalence was significantly lower than that measured in BC (Figure 3, Table 1). Neither HER2 overexpression nor HER2 amplification was observed in DM skin biopsies (data not shown).

Programmed death-ligand 1 (PD-L1) was clearly detectable in more than 10% of BC cells, which showed a predominant diffuse pattern of expression (Figure 3). This finding resulted in a PD-L1 expression score (H score) of 80 (range 0–300) (9, 10). Normal, ER-positive, mammary gland at the periphery of the tumor mass also displayed PD-L1 overexpression (Figure 3). The same analysis on the skin biopsy, obtained at the time of overt DM, showed that PD-L1 was greatly expressed by the basal layer of epidermal cells and, to a more extent, by dermal adnexa (Figure 3).

The documented expression of PD-L1 in the spared mammary gland (SMG) within the tumor sample prompted us to investigate the immune contexture in these structures (Figure 3). Similar to what observed in DM skin samples, most residual mammary tissue located along the tumor invasive margin was highly infiltrated by T-lymphocytes (i.e., breast tissue-infiltrating lymphocytes, BILs) displaying a cytotoxic CD8+ phenotype and low expression of PD-1 (PD-1/CD8 ratio in SMG: 0.54) (Figure 3, Table 1). Immune cells were absent in tumor distant, PD-L1-positive, mammary structures. The evaluation of the skin biopsy after clinical remission of DM clearly documented an attenuation of PD-L1 expression and the disappearance of CD8 driven immune reaction. These findings are representative of a “cold” skin tissue where no immune cells could be found (11).

## Discussion

In this brief report, we present the peculiar case of a HER2-positive early BC patient with synchronous DM, which persisted after tumor surgical removal, and disappeared after the first administration of adjuvant CIT with trastuzumab.

DM is an autoimmune disease consisting of a chronic inflammatory injury of striated muscle and skin with an incidence of 1/100,000 (4). Approximately 15–30% of DMs are associated with underlying malignancies (4). Although DM may be a systemic manifestation of cancer, is not the consequence of a skin tumor invasion (12).

Our immunological analyses showed the presence of immune cell infiltrates in both patient's tumor and skin specimens, which shared the same predominant CD3+ T cell phenotype. Furthermore, the patient presented remarkable levels of natural serum anti-HER2 antibodies, which persisted 16 months after the last administration of trastuzumab. Previous studies have documented that the presence of TILs infiltration and the increased endogenous anti-HER2 antibody immunity following adjuvant CIT with trastuzumab are associated with improved disease-free survival (DFS) (13–15).

Our findings confirm the induction of a HER2-specific adaptive immune response and support the hypothesis that HER2-specific T-cell clones cross-reacted with one or more unknown skin antigens, causing DM. Noteworthy, SILs and TILs were qualitatively different: although keratinocytes were PDL1-positive, the predominant CD8+ cytotoxic infiltrate in DM skin showed low PD-1 expression, thus avoiding immune tolerance. Conversely, at tumor site, PD-L1-positive cancer cells were associated with predominant CD4+ TIL phenotype and high PD-1 expression, suggesting the induction of immune tolerance. These data confirm the association between PD-1/PD-L1 axis and HER2 signaling (16, 17), and give the rationale for anti-HER2 plus anti-PD-1/PD-L1 combination strategies. In this context, the KATE-2 trial has recently showed that atezolizumab (anti-PD-L1) plus trastuzumab emtansine (T-DM1) may improve overall survival in PD-L1-positive, HER2-positive advanced BC (18). Interestingly, the normal mammary gland also displayed PD-L1 expression, which is usually upregulated in response to inflammatory or auto-immune processes (19). Similar to what observed in DM skin samples, a CD8+, PD-1^low^ immune infiltrate was found in the PD-L1-expressing, cancer-spared mammary gland located along the tumor invasive margin. Conversely, distal PD-L1-positive mammary structures did not contain immune cells, and skin samples obtained after the clinical remission of DM showed low PD-L1 expression and disappearance of CD8+ T cells.

In our clinical case, the immune over-activation observed before treatment may have limited the cancer spreading but, at the same time, induced DM. Neither HER2-overexpression or HER2-positive tumor cells were identified in the skin tissue. According to these findings, we hypothesize that HER2-specific T-cell clones, which were induced by the over-expression of the HER2 antigen in the primary BC, cross-reacted with one or more unknown skin antigens, causing DM. The adjuvant administration of CIT with trastuzumab may have been able to silence skin cross-reaction through its ability to eliminate any residual HER2-positive micrometastatic disease, wherever it was found in the body, thus leading to DM remission.

## Conclusion

The clinico-pathological characteristics of the present case and the immunological analyses performed on patient tumor, skin, and blood samples suggest a proof of principle for a unique pathogenesis underlying the coexistence of cancer and autoimmune disease. The findings here reported may also help address diagnostic and therapeutic issues in autoimmune disorders associated with anti-PD-1/PD-L1 therapies.

## Data Availability Statement

All datasets generated for this study are included in the article/supplementary material.

## Ethics Statement

The studies involving human participants were reviewed and approved by Comitato Etico della Provincia di Parma. The patients/participants provided their written informed consent to participate in this study. Written informed consent was obtained from the individual(s) for the publication of any potentially identifiable images or data included in this article.

## Author Contributions

AM and BP conceived of the presented idea. FQ and GMi planned the experiments. GMa, CM, CL, and DM carried out the experiments. BP, AM, FQ, GMi, GMa, and CL contributed to the interpretation of the results. BP took the lead in writing the manuscript. AM revised the manuscript. All authors provided critical feedback and helped shape the research, analysis and manuscript.

### Conflict of Interest

The authors declare that the research was conducted in the absence of any commercial or financial relationships that could be construed as a potential conflict of interest.

## References

[1] Slamon DJ Clark GM Wong SG Levin WJ Ullrich A McGuire WL. Human breast cancer: correlation of relapse and survival with amplification of the HER-2/neu oncogene. Science. (1987) 235:177–82. 3798106 10.1126/science.3798106

[2] Musolino A Boggiani D Sikokis A Rimanti A Pellegrino B Vattiato R. Prognostic risk factors for treatment decision in pT1a,b N0M0 HER2-positive breast cancers. Cancer Treat Rev. (2016) 43:1–7. 10.1016/j.ctrv.2015.11.010 26827687

[3] Bianchini G Gianni L. The immune system and response to HER2-targeted treatment in breast cancer. Lancet Oncol. (2014) 15:e58–68. 10.1016/S1470-2045(13)70477-7 24480556

[4] Callen JP Hyla JF Bole GG Kay DR. The relationship of dermatomyositis and polymyositis to internal malignancy. Arch Dermatol. (1980) 116:295–8. 7369745

[5] Caproni M Torchia D Cardinali C Volpi W Del Bianco E D'Agata A. Infiltrating cells, related cytokines and chemokine receptors in lesional skin of patients with dermatomyositis. Br J Dermatol. (2004) 151:784–91. 10.1111/j.1365-2133.2004.06144.x 15491417

[6] Miller FW Cooper RG Vencovský J Rider LG Danko K Wedderburn LR. Genome-wide association study of dermatomyositis reveals genetic overlap with other autoimmune disorders. Arthritis Rheum. (2013) 65:3239–47. 10.1002/art.38137 23983088 PMC3934004

[7] Hill CL Zhang Y Sigurgeirsson B Pukkala E Mellemkjaer L Airio A. Frequency of specific cancer types in dermatomyositis and polymyositis: a population-based study. Lancet. (2001) 357:96–100. 10.1016/S0140-6736(00)03540-6 11197446

[8] Salgado R Denkert C Campbell C Savas P Nuciforo P Aura C Tumor-infiltrating lymphocytes and associations with pathological complete response and event-free survival in HER2-positive early-stage breast cancer treated with lapatinib and trastuzumab: a secondary analysis of the NeoALTTO Trial. JAMA Oncol. (2015) 91:165–71. 10.1016/j.chemosphere.2012.12.037.Reactivity PMC5551492 26181252

[9] Hirsch FR Varella-Garcia M Bunn PA Di Maria M V Veve R Bremmes RM. Epidermal growth factor receptor in non-small-cell lung carcinomas: correlation between gene copy number and protein expression and impact on prognosis. J Clin Oncol. (2003) 21:3798–807. 10.1200/JCO.2003.11.069 12953099

[10] John T Liu G Tsao M-S. Overview of molecular testing in non-small-cell lung cancer: mutational analysis, gene copy number, protein expression and other biomarkers of EGFR for the prediction of response to tyrosine kinase inhibitors. Oncogene. (2009) 28:S14–23. 10.1038/onc.2009.197 19680292

[11] Planes-Laine G Rochigneux P Bertucci F Chrétien AS Viens P Sabatier R. PD-1/PD-L1 targeting in breast cancer: the first clinical evidences are emerging. A literature review. Cancers. (2019) 11:1033. 10.3390/cancers11071033 31336685 PMC6679223

[12] Hendren E Vinik O Faragalla H Haq R. Breast cancer and dermatomyositis: a case study and literature review. Curr Oncol. (2017) 24:e429–33. 10.3747/co.24.3696 29089813 PMC5659167

[13] Dieci M V Prat A Tagliafico E Paré L Ficarra G Bisagni G. Integrated evaluation of PAM50 subtypes and immune modulation of pCR in HER2-Positive breast cancer patients treated with chemotherapy and HER2-Targeted agents in the CherLOB trial. Ann Oncol. (2016) 27:1867–73. 10.1093/annonc/mdw262 27484801

[14] Dieci MV Mathieu MC Guarneri V Conte P Delaloge S Andre F. Prognostic and predictive value of tumor-infiltrating lymphocytes in two phase III randomized adjuvant breast cancer trials. Ann Oncol. (2015) 26:1698–704. 10.1093/annonc/mdv239 25995301 PMC4511223

[15] Norton N Fox N McCarl C-A Tenner KS Ballman K Erskine CL. Generation of HER2-specific antibody immunity during trastuzumab adjuvant therapy associates with reduced relapse in resected HER2 breast cancer. Breast Cancer Res. (2018) 20:52. 10.1186/s13058-018-0989-8 29898752 PMC6000975

[16] Zampieri S Valente M Adami N Biral D Ghirardello A Rampudda ME. Polymyositis, dermatomyositis and malignancy: a further intriguing link. Autoimmun Rev. (2010) 9:449–53. 10.1016/j.autrev.2009.12.005 20026430

[17] Bertucci F Gonçalves A. Immunotherapy in breast cancer: the emerging role of PD-1 and PD-L1. Curr Oncol Rep. (2017) 19:64. 10.1007/s11912-017-0627-0 28799073

[18] Emens LA Esteva FJ Beresford M Saura C De Laurentiis M Kim S-B 305O. Overall survival (OS) in KATE2, a phase II study of programmed death ligand 1 (PD-L1) inhibitor atezolizumab (atezo)+trastuzumab emtansine (T-DM1) vs placebo (pbo)+T-DM1 in previously treated HER2+ advanced breast cancer (BC). Ann Oncol. (2019) 30:v104 10.1093/annonc/mdz242

[19] Sharpe AH Wherry EJ Ahmed R Freeman GJ. The function of programmed cell death 1 and its ligands in regulating autoimmunity and infection. Nat Immunol. (2007) 8:239–45. 10.1038/ni1443 17304234

